# Cell Division Cycle 5-Like Regulates Metaphase-to-Anaphase Transition in Meiotic Oocyte

**DOI:** 10.3389/fcell.2021.671685

**Published:** 2021-07-01

**Authors:** Hong-Yong Zhang, Jian Li, Ying-Chun Ouyang, Tie-Gang Meng, Chun-Hui Zhang, Wei Yue, Qing-Yuan Sun, Wei-Ping Qian

**Affiliations:** ^1^Department of Reproductive Medicine, Peking University Shenzhen Hospital, Shenzhen Peking University-The Hong Kong University of Science and Technology Medical Center, Shenzhen, China; ^2^State Key Laboratory of Stem Cell and Reproductive Biology, Institute of Zoology, Chinese Academy of Sciences, Beijing, China; ^3^Guangdong Key Laboratory of Male Reproductive Medicine and Genetics, Shenzhen PKU-HKUST Medical Center, Institute of Urology, Peking University Shenzhen Hospital, Shenzhen, China; ^4^Fertility Preservation Lab, Reproductive Medicine Center, Guangdong Second Provincial General Hospital, Guangzhou, China

**Keywords:** meiotic progression, Cdc5L, securin, APC/C, mouse oocyte

## Abstract

The quality of oocytes is a vital factor for embryo development. Meiotic progression through metaphase I usually takes a relatively long time to ensure correct chromosome separation, a process that is critical for determining oocyte quality. Here, we report that cell division cycle 5-like (Cdc5L) plays a critical role in regulating metaphase-to-anaphase I transition during mouse oocyte meiotic maturation. Knockdown of Cdc5L by small interfering RNA injection did not affect spindle assembly but caused metaphase I arrest and subsequent reduced first polar body extrusion due to insufficient anaphase-promoting complex/cyclosome activity. We further showed that Cdc5L could also directly interact with securin, and Cdc5L knockdown led to a continuous high expression level of securin, causing severely compromised meiotic progression. The metaphase-to-anaphase I arrest caused by Cdc5L knockdown could be rescued by knockdown of endogenous securin. In summary, we reveal a novel role for Cdc5L in regulating mouse oocyte meiotic progression by interacting with securin.

## Introduction

In mammals, germ cell undergoes two continuous cell divisions to generate haploid gametes. Homologous chromosomes are separated during the first meiosis, and sister chromatids are separated during the second meiosis in the oocyte (Meng et al., 2020). At metaphase I, chromosomes are attached by cohesin, an evolutionary conserved multiprotein complex including several core subunits: structural maintenance of chromosomes 1 beta, structural maintenance of chromosomes 3 (SMC3), REC8 meiotic recombination protein, RAD21 cohesin complex component like, and cohesin subunit SA-3 (Ishiguro, 2019). At anaphase I, separase (also known as extra spindle pole bodies like 1) cleaves the cohesin complex, which locates on the chromosome arms, leading to homologous chromosome segregation. During meiosis II, separase cleaves centromeric cohesin to ensure proper sister chromatid segregation (Kitajima et al., 2006). Separase activity is regulated by a separase inhibitor, securin (also known as PTTG1) (Petronczki et al., 2003). During the metaphasetoanaphase transition, the anaphase-promoting complex/cyclosome (APC/C) takes on the responsibility for degradation of securin to release the inhibition of seperase activity, leading to the destruction of the cohesion complex (Nabti et al., 2017; Li et al., 2019). APC/C is a complex containing E3 ubiquitin ligase that controls the degradation of cyclin B1 (CCNB1) and securin during the metaphasetoanaphase transition in all eukaryotes (Yamano, 2019). Although the Cdc20 and Cdh1 have been confirmed as co-activators whereas Emi2 as an inhibitor of APC/C, the regulators and substrates of APC/C have not been totally identified (Yamano, 2019). Nabti et al. (2008) showed that securin but not CDK1/cyclin B1 regulates sister chromatid disjunction during meiosis II in mouse eggs. Moreover, they found that separase activity is most likely regulated by securin rather than by CDK1/cyclin B1. We recently showed that the cyclin B2/CDK1 complex is a key controller to regulate chromosome separation by inhibitory phosphorylation of separase activity in mouse oocytes (Li et al., 2019). Nevertheless, the metaphasetoanaphase transition involves a complicated signal network that needs further clarification.

Cell division cycle 5-like (Cdc5L) is a core component protein in the pre-messenger RNA (mRNA) processing factor 19 associated complex, which is a putative E3 ubiquitin ligase complex and is functional in pre-mRNA processing and DNA damage response (Zhang et al., 2009; Huang et al., 2017). Previous studies have shown that Cdc5L contributed to the progression of various cancers, such as colorectal cancer (Li et al., 2017), prostate cancer (Li et al., 2018), gliomas (Chen et al., 2016), bladder cancer (Zhang et al., 2020), and hepatocellular carcinoma (Qiu et al., 2016). It was reported that Cdc5L is a cell cycle regulator gene to promote mitotic progression, and depletion of Cdc5L causes obvious chromosome misalignment, sustained activation of spindle assembly checkpoint (SAC), and mitotic arrest (Mu et al., 2014). Another report showed that Cdc5L interacts with NIPP1, a regulatory subunit of protein phosphatase-1 (PP1), but it does not regulate G2/M transition in Hela cells (Boudrez et al., 2000). Liu et al. (2018) first reported that Cdc5L is highly expressed in the porcine oocyte, and they found that knockdown of Cdc5L reduced the rate of germinal vesicle break down (GVBD) and polar body extrusion (PBE), as well as induced abnormal chromatin. In addition, they further found that Cdc5L is crucial for early embryo development in porcine. However, no details have been reported regarding the phenotypes in oocyte meiotic maturation, and no mechanistic studies have been performed.

Here, we showed that knockdown of Cdc5L decreased the oocyte maturation rate and that oocytes failed to enter anaphase due to the reduced activity of APC/C. Cdc5L knockdown-induced meiotic metaphase I arrest was SAC independent but was due to the failed degradation of securin. The metaphase I arrest induced by Cdc5L knockdown could be rescued by securin knockdown by small interfering RNA (siRNA) injection. Moreover, we showed that Cdc5L could also interact directly with securin. We confirmed that Cdc5L is a critical factor of metaphasetoanaphase I transition in mouse oocyte meiotic progression.

## Materials and Methods

### Oocyte Collection and Culture

All experiments involving animals were approved by the Guidelines for the Use of Animals issued by the Institute of Zoology, Chinese Academy of Sciences. The 6–8-week-old ICR female mice were killed for oocyte collection 48 h after intraperitoneal injection of 10-IU pregnant mare serum gonadotropin to promote follicular maturation. Then, immature germinal vesicle (GV) oocytes, with a diameter of approximately 80 μm and centrally located germinal vesicle, were collected and cultured in M2 medium (Sigma) supplemented with 200-μM 3-isobutyl-1-methylxanthine (IBMX) to prevent GVBD. After 24 h in IBMX, the oocytes were placed in IBMX-free M2 medium under liquid paraffin oil at 37°C in an atmosphere of 5% carbon dioxide in the air for further culture.

### RNA Extraction and Real-Time Quantitative Polymerase Chain Reaction Analysis

The mRNA was extracted using a Dynabeads^TM^ mRNA DIRECT^TM^ Micro kit (Invitrogen, 61021) from 30 oocytes in each group. Then, complementary DNA (cDNAs) were synthesized by 5 × All-In-One RT MasterMix kit (abm, G490). Real-time quantitative polymerase chain reaction (RT-qPCR) was conducted with *Cdc5L*-specific primers as follows: forward: 5′-GCCTGACCCGATAGACATGG-3′; reverse: 5′-TTGAGTATTGGCCAGACGGG-3′. *Gapdh* represented the reference gene amplification using the following primers: forward: 5′-CCCCAATGTGTCCGTCGTG-3′; reverse: 5′-TGCCTGCTTCACCACCTTCT-3′. Preparation of a total volume of 20 μl, including the primer, cDNA template, and EvaGreen 2 × qPCR MasterMix-No Dye (abm, MasterMix-S). The PCR was performed by Roche Light Cycler 480 and using the following procedure: 1 min at 95°C, followed by 40 cycles of 15 s at 95°C, 15 s at 60°C, and 45 s at 72°C, and the melting curve conditions were 1 min at 95°C, 30 s at 60°C, and 30 s at 95°C. Actin gene as a reference and relative gene expression was analyzed based on the 2^–ΔΔCt^ method.

### Microinjection of Small Interfering RNA or Messenger RNA

siRNA was used for *Cdc5L* knockdown (sc62089, Santa Cruz Biotechnology), and siRNA at a concentration of 25 μM was injected into the GV oocytes by an Eppendorf microinjector (*Pi* = 150; *t* = 0.5s; *Pc* = 30). The same amount of negative control siRNA was used as control. After 24 h of culture in M2 medium with 200-μM IBMX, the oocytes were further cultured in IBMX-free medium at a condition of 5% carbon dioxide, 37°C, and saturated humidity condition for an additional 12 h to observe the rate of PBE. The separase sensor, *mCherry-Securin*, and *Ccnb1-Venus* plasmid were previously described by Li et al. (2019). The Cdc5L cDNA was cloned into a pCDNA3.1-EGFP plasmid and then was transcribed *in vitro* by mMESSAGE mMACHINE T7 (Invitrogen, AM1344).

### Co-immunoprecipitation and Western Blotting

The Myc-Cdc5L and EGFP-securin plasmids were constructed for immunoprecipitation (IP). According to the previous description, the plasmids were transfected into 293T (Li et al., 2019). After 48 h, we collected the cells with 400-μl IP lysis buffer (Thermo Fisher Scientific, 88804) with the cocktail of protease and phosphatase inhibitors, followed by incubation on ice for 20 min. Next, we centrifuged the lysates at 13,400 rpm for 10 min at 4°C, and 400 μl of the supernatant was incubated with 1-μg primary antibody (GFP: abcam, ab290 or Myc: sigma, M4439) and 20-μl Magnetic Beads (Thermo Fisher Scientific, 88804) at 4°C overnight with rotation. The next morning, after washing five times, 40-μl 1 × sodium dodecyl sulfate loading buffer was added to the immunoprecipitate, and Western blotting was prepared. Western blot experiment was performed as described previously (Zhang et al., 2021). The anti-β-actin antibody (ZSGB-BIO, TA-09,1:1,000), rabbit anti-Cdc5L antibody (Thermo, 702831, 1:500), mouse monoclonal anti-Myc antibody (Sigma, M4439, 1:3,000), mouse monoclonal anti-Cyclin B1 antibody (Abcam, ab72, 1:1,000), and anti-SMC3 antibody (Abcam, ab128919, 1:1,000) were used for Western blotting.

### Immunofluorescence Analysis

For spindle staining, oocytes were first fixed in 4% paraformaldehyde in phosphate-buffered saline (PBS) buffer for at least 30 min at room temperature and permeabilized with 0.5% Triton X-100 for 20 min. The oocytes were then blocked in PBS containing 2% bovine serum albumin for 1 h at room temperature. The oocytes were incubated overnight at 4°C with 1:800 anti-α-tubulin–fluorescein isothiocyanate antibody (322588, Thermo Fisher Scientific). Next, oocytes were washed three times with PBS containing 0.1% Tween-20 and 0.01% Triton X-100, and DNA was stained with 4′,6-diamidino-2-phenylindole (DAPI) (1 μg/ml in PBS; Sigma-Aldrich). The samples were mounted on glass slides and were observed under a laser-scanning confocal microscope (Zeiss LSM 780).

### Chromosome Spreads

The zona pellucida of the oocyte was removed by treatment with Acid Tyrode’s solution (Sigma-Aldrich) at room temperature. Then, the oocytes were placed onto glass slides and fixed in a solution described by Li et al. (2019). The samples were placed in a humidifying box at room temperature for several hours, and then 2% bovine serum albumin was used for blocking at room temperature. The oocytes were then incubated with an anti-Bub3 antibody (1:200; ab133699, Abcam) and an anti-SMC3 antibody (1:20; ab128919, Abcam). After washing three times with washing buffer, the oocytes were incubated with the secondary antibody at room temperature for 1 h. Finally, 1 μg/ml DAPI was used to visualize the chromosomes.

### Time-Lapse Live Imaging Experiments

After microinjecting *Cdc5L* siRNA, the oocytes were cultured in M2 medium supplemented with 200-μM IBMX for 24 h; separase sensor mRNA, *mCherry-Securin*, or *Ccnb1-Venus* mRNA was injected into the oocytes. After mRNA injection, GV oocytes were cultured in an M2 medium with IBMX for 2 h to allow mRNA expression. Time-lapse imaging was performed by a spinning disk confocal microscope imaging system (Andor Dragfly 200). The images of oocytes were acquired every 30 or 10 min. Finally, the results were analyzed by Imaris.

### Statistical Analysis

Statistical analysis was conducted using Prism Software (GraphPad). For statistical comparison, Student’s *t*-test was used to compare between two groups. A *P* < 0.05 was considered significant. For every experiment, at least three replications were conducted.

## Results

### Expression and Subcellular Localization of Cell Division Cycle 5-Like During Mouse Oocyte Maturation

**FIGURE 1:**
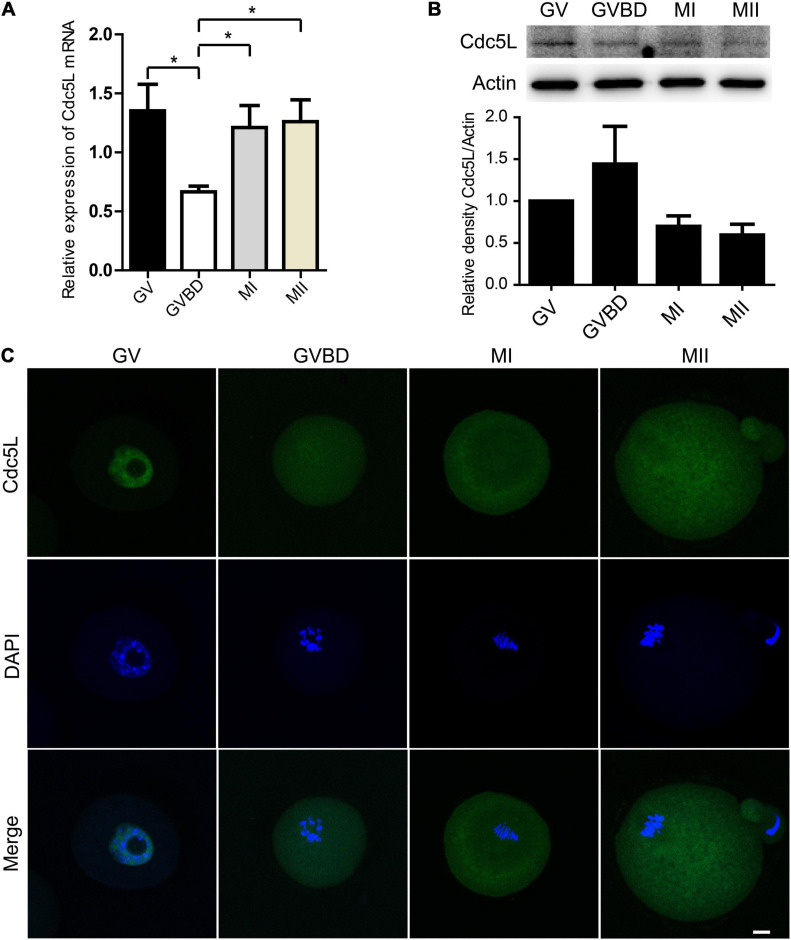
Expression and subcellular localization of Cdc5L during mouse oocyte meiotic maturation. **(A)** Relative level of *Cdc5L* mRNA identified by RT–PCR. A total of 50 oocytes were collected for each sample. At least three replications were conducted, and 150 oocytes were used for each group. Data are shown as means ± SEM. “*” represents *P* < 0.05; “**” represents *P* < 0.01. **(B)** Expression of Cdc5L protein revealed by Western blotting analysis. A total of 150 oocytes were collected after culture for 0, 4, 8, and 12 h; time points when most oocytes had reached GV, GVBD, MI, and MII stages, respectively. At least three replications were conducted, and 450 oocytes were used for each group. β-actin is shown as an internal control. **(C)** Subcellular localization of Cdc5L as revealed by immunofluorescence staining. Confocal microscopy images that showed subcellular localization of Cdc5L (green) and DNA (blue) was counterstained with DAPI. Scale bars: 10 μm.

To investigate the role of Cdc5L during oocyte meiosis I, firstly, we detected the expression of Cdc5L. Oocytes at different stages were collected, including GV, GVBD, metaphase I (MI), and metaphase II (MII), respectively, corresponding to culture for 0, 4, 8, and 12 h, and then the *Cdc5L* mRNA level was detected by RT-qPCR. The results showed that *Cdc5L* mRNA was expressed at the four stages, and at the GVBD stage, its expression was significantly lower than in other stages of mouse oocyte maturation (Figure 1A). This is not surprising, as a recent study showed that nuclear envelope fluctuations modulate oocyte transcriptome and regulate gene expression in fully grown oocytes (Almonacid et al., 2019), although it is generally regarded that the nucleus of the fully grown oocyte is transcriptionally silent. The Western blots assay showed that Cdc5L protein was relatively lower in MI and MII stages than in GV and GVBD stages (Figure 1B). To validate the subcellular-specific distribution of Cdc5L, we injected 500 ng/ml *Cdc5L-EGFP* mRNA into oocytes at the GV stage. Immunofluorescence staining showed that Cdc5L-EGFP concentrated in the nucleus at the GV stage and mainly distributed in the cytoplasm from the GVBD stage to the MII stage (Figure 1C).

### Knockdown of Cell Division Cycle 5-Like Does Not Affect Germinal Vesicle Breakdown but Causes Decreased PB1 Extrusion

**FIGURE 2:**
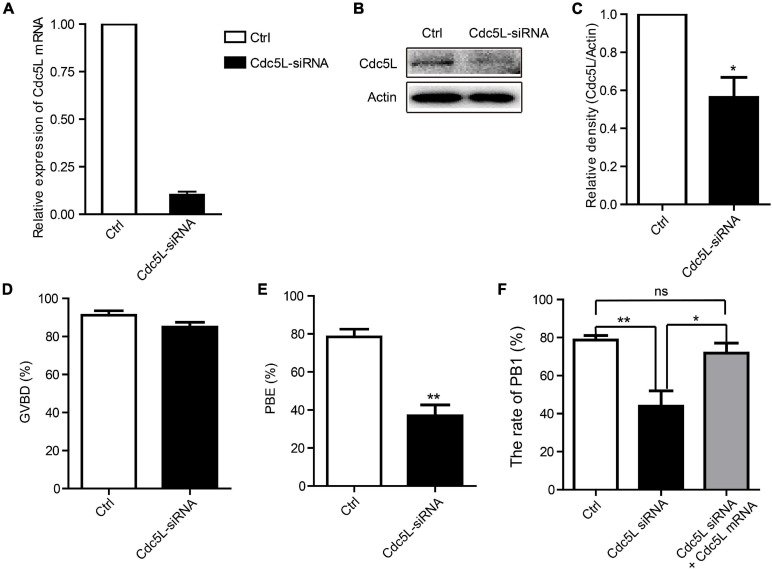
Knockdown of Cdc5L reduces rate of PB1 extrusion. **(A)** Relative level of Cdc5L mRNA identified by RT–PCR in control and Cdc5L siRNA oocytes. A total of 50 oocytes were collected in each group after culture for 24 h in M2 medium with IBMX. At least three replications were conducted, and 150 oocytes were used for each group. Data are shown as means ± SEM. “*” represents *P* < 0.05; “**” represents *P* < 0.01. **(B)** Western blotting results for Cdc5L and β-actin in Cdc5L-siRNA injected oocytes and control oocytes after culture for 24 h in M2 medium with IBMX (150 oocytes per sample). At least three replications were conducted, and 450 oocytes were used for each group. **(C)** Relative staining intensity of Cdc5L assessed by densitometry. Data are shown as means ± SEM. “*” represents *P* < 0.05; “**” represents *P* < 0.01. At least three replications were conducted. **(D)** Percentages of GVBD were quantified in control and Cdc5L siRNA oocytes. Data are shown as means ± SEM. “*” represents *P* < 0.05; “**” represents *P* < 0.01. At least three replications were conducted. **(E)** Rate of PB1 extrusion was quantified in control and Cdc5L siRNA oocytes. Data are shown as means ± SEM. “*” represents P < 0.05; “**” represents *P* < 0.01. At least three replications were conducted. **(F)** Rates of PB1 extrusion were quantified in control, Cdc5L siRNA, and Cdc5L mRNA + siRNA oocytes. Data are shown as means ± SEM. “*” represents *P* < 0.05; “**” represents *P* < 0.01. At least three replications were conducted.

To investigate the functions of Cdc5L in oocyte meiotic progression, we knocked down *Cdc5L* by injecting specific siRNA. The GV stage oocytes were collected from 8-week-old female mice, and after *Cdc5L*-specific or negative control siRNA injection, the oocytes were cultured in an M2 medium containing 200-μM IBMX for 24 h to deplete the protein. Then, the protein expression of Cdc5L siRNA oocytes was verified by Western blotting, which showed that Cdc5L knockdown was effective (Figures 2A–C). Then, the injected oocytes were released from IBMX for an additional 14 h further observation. Unexpectedly, we found that knockdown did not affect the development rate of GVBD in both Cdc5L-siRNA group and control group (84.92 ± 2.56%, *n* = 163 vs. 91.19 ± 2.39%, *n* = 151; *P* = 0.10) (Figure 2D). However, the first polar body (PB1) extrusion rate (36.94 ± 5.69%, *n* = 179) in the Cdc5L siRNA group was significantly reduced compared with control group (78.47 ± 4.06%, *n* = 129) (Figure 2E). The phenotype was rescued by overexpressing Cdc5L mRNA to exclude the possibility of off-target effects (Figure 2F). These results indicate that Cdc5L-knockdown oocytes are arrested at the stage between the GVBD and PB1 extrusion, generally called metaphase I arrest.

### Cell Division Cycle 5-Like Knockdown Does Not Affect Meiotic Spindle Assembly

To further investigate whether oocyte meiotic arrest is caused by disrupted meiotic spindle organization, we next examined the morphology of spindles. After *Cdc5L* or control siRNA injection, the oocytes were inhibited at the GV stage for 24 h and then released from IBMX for 8 h further culture. Then, the oocytes were collected for immunofluorescence with an anti-α-tubulin–fluorescein isothiocyanate antibody to observe the spindle and counterstained with DAPI to visualize the chromosome alignment. Our results showed that in the Cdc5L-siRNA group, oocytes exhibited normal morphological spindles as the same as the control group (Supplementary Figure 1). When examining the meiotic spindles in oocytes that failed to extrude the PB1 after extended culture, we found that oocytes at the MI stage exhibited normal spindles (data not shown). These results suggest that knockdown of Cdc5L does not affect spindle assembly but results in metaphase I arrest during mouse oocyte maturation.

### Separase Keeps Inactive During Meiosis I in Cell Division Cycle 5-Like Knockdown Oocytes

**FIGURE 3:**
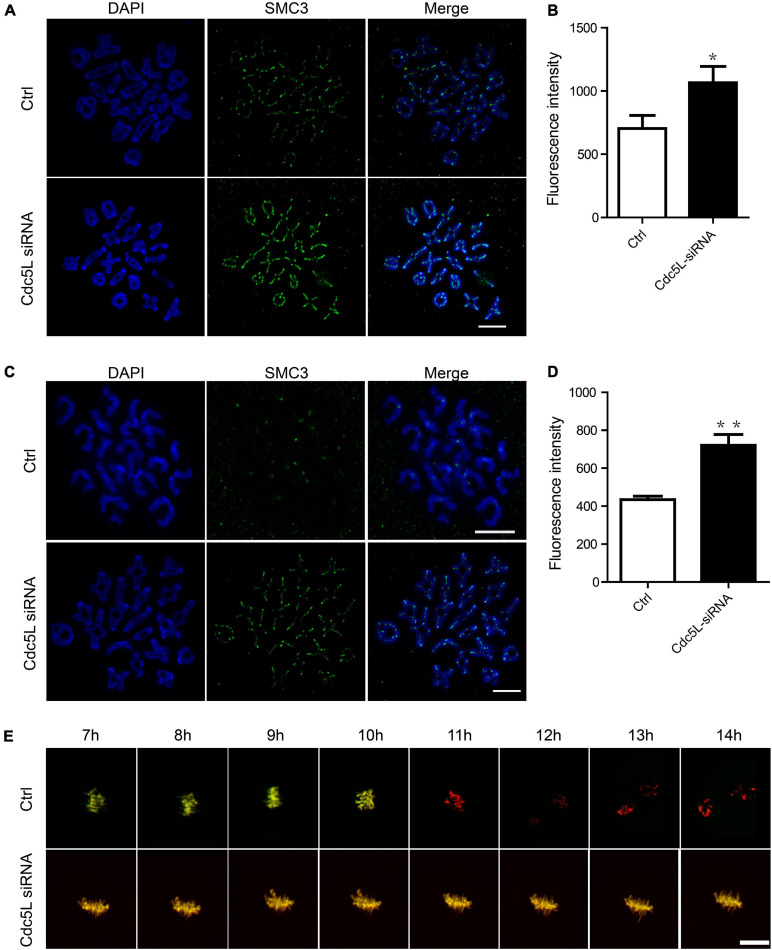
Activity of separase during meiosis I in mouse oocytes. **(A)** Chromosome spread and SMC3 detection in control (*n* = 18) and Cdc5L knockdown (*n* = 20) oocytes. Oocytes were collected at 10 after release from IBMX. ACA, anti-centromeric antibody. Scale bars: 10 μm. **(B)** Analysis of SMC3 density in oocytes at 10 h after release from IBMX. Data are shown as means ± SEM. “*” represents *P* < 0.05; “**” represents *P* < 0.01. At least three replications were conducted. Oocytes were analyzed for each group as follows: Ctrl, *n* = 18; Cdc5L siRNA, *n* = 20. **(C)** Chromosome spread and SMC3 detection in control (*n* = 21) and Cdc5L knockdown (*n* = 20) oocytes. Oocytes were collected at 14 after release from IBMX. ACA, anti-centromeric antibody. Scale bars: 10 μm. **(D)** Analysis of SMC3 density in oocytes at 14 h after release from IBMX. Data are shown as means ± SEM. “*” represents *P* < 0.05; “**” represents *P* < 0.01. At least three replications were conducted. Oocytes were analyzed for each group as follows: Ctrl, *n* = 21; Cdc5L siRNA, *n* = 20. **(E)** Representative time-lapse confocal images of separase sensor in control and Cdc5L knockdown oocytes. Concentration of separase sensor mRNA used was 200 ng/μl. Scale bars: 30 μm. Oocytes were analyzed for each group as follows: Ctrl, *n* = 15; Cdc5L siRNA, *n* = 13.

As previously reported, separase is responsible for chromosome separation. In general, separase cleavages of the arm cohesin while the centromeric cohesin is protected during meiosis I. The separase activity is inhibited before metaphase I, and its activity is released timely during the metaphase–anaphase I transition. To evaluate the activity of separase, we first determined the expression of SMC3, a subunit of cohesion, at 10 h of culture (metaphase–anaphase I transition) and 14 h of culture in Cdc5L knockdown and control oocytes. We found the total integrity of SMC3 expression on chromosome arms in Cdc5L knockdown oocytes at 10 h; in contrast, SMC3 was removed mostly from chromosome arms in the control oocytes (Figure 3A). Besides, the fluorescence intensity of SMC3 was significantly higher in the Cdc5L knockdown oocytes cultured for 10 h (Figure 3B). Furthermore, we also found that bivalents (MI) were maintained well in most of the Cdc5L knockdown oocytes at 14 h, whereas univalent chromosomes (MII) were observed in most control oocytes (Figure 3C), and fluorescence intensity of SMC3 on kinetochores was significantly higher in the Cdc5L knockdown oocytes (Figure 3D).

To test the separase activity directly, we injected a separase sensor mRNA mixture with *Cdc5L* siRNA or negative control siRNA into the GV oocytes and cultured it for 24 h in M2 with 200-μM IBMX. Then, the oocytes were released from IBMX and were used for the live-cell confocal assay. A Separase sensor is a method for detecting separase activity in mouse oocytes *in vivo*. This method utilizes an H2B-mCherry fused with RAD21-EGFP as described by our previous study (Li et al., 2019). RAD21 is a substrate of separase and can be cleaved by separase. The sensor is loaded on the chromosomes through its H2B-tag, and the signals from both mCherry and EGFP are visible. Upon separase activation, the RAD21 fragment is cleaved, and EGFP signal disappears from the chromosomes. The change between mCherry and EGFP fluorescence is a readout of separase activity. The EGFP signal disappeared suddenly on chromosomes in control oocytes, whereas the EGFP signal was maintained consistently on chromosomes in Cdc5L knockdown oocytes during meiosis I (Figure 3E). Taken together, these results clearly demonstrate that Cdc5L knockdown impairs separase activity in mouse oocyte meiosis I.

### Knockdown of Cell Division Cycle 5-Like Induces Insufficient Anaphase-Promoting Complex/Cyclosome Activity Independent of SAC

**FIGURE 4:**
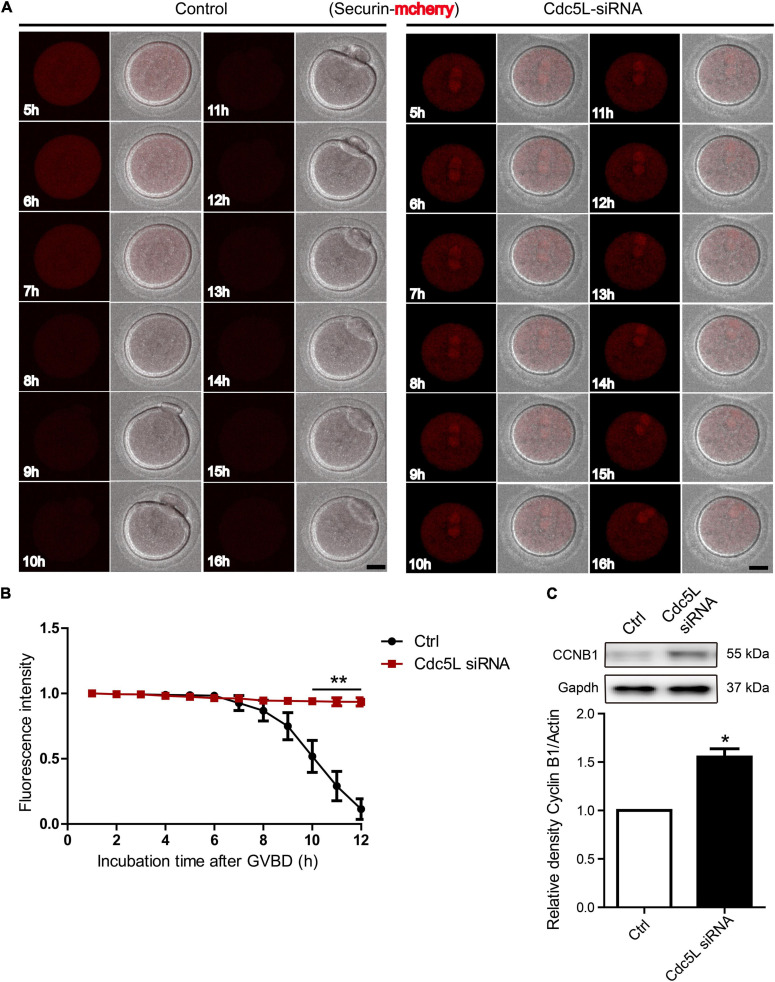
Knockdown of Cdc5L induces insufficient APC/C activity. **(A)** Representative time-lapse confocal images for mCherry-securin in control (*n* = 20) and Cdc5L knockdown (*n* = 15) oocytes. Concentration of separase sensor mRNA used was 200 ng/μl. Scale bars: 20 μm. **(B)** Analysis of mCherry-securin fluorescence intensity in control and Cdc5L knockdown oocytes. Data are shown as means ± SEM. “*” represents *P* < 0.05; “**” represents *P* < 0.01. Oocytes were analyzed for each group as follows: Ctrl, *n* = 20; Cdc5L siRNA, *n* = 15. **(C)** Expression of CCNB1 protein as revealed by Western blotting analysis. Oocytes were collected at 9.5 h after release from IBMX (150 oocytes per sample). At least three replications were conducted, and 450 oocytes were used for each group.

To illustrate the reason for separase inhibition, we asked whether securin, which inhibited separase activity, was degraded in Cdc5L knockdown oocytes. A live-cell confocal system was used for detecting the expression of securin-mCherry-labeled *Securin* mRNA, and *Cdc5L* siRNA or negative control siRNA was co-injected into the GV oocytes. The oocytes were inhibited for 24 h, and then the oocytes were released from IBMX for confocal live-cell imaging observation. We found that the red fluorescence intensity remained almost unchanged in the Cdc5L knockdown oocytes, whereas the control oocytes experienced a significant decline with PB1 extrusion (Figures 4A,B and Supplementary Movie 1).

**FIGURE 5:**
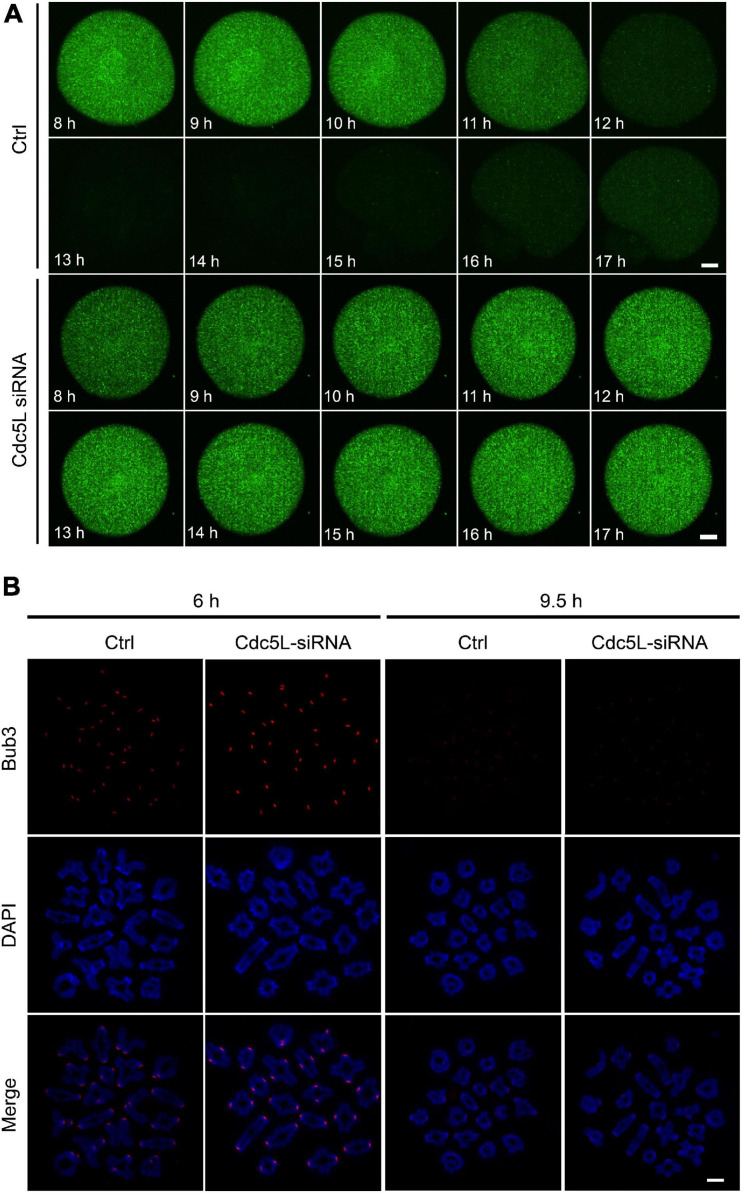
Knockdown of Cdc5L does not affect normal function of SAC. **(A)** Time-lapse fluorescence measurement of CCNB1-EGFP after low concentration mRNA injection at GV stage. 20 ng/μl *Ccnb1*-*EGFP* mRNA was used for injection. Oocytes were analyzed for each group as follows: Ctrl, *n* = 8; Cdc5L siRNA, *n* = 7. Scale bars: 10 μm. **(B)** Chromosome spreads at 6 h (pro-Met I) (Ctrl, *n* = 8; Cdc5L siRNA, *n* = 10) and 9.5 h (Met I) (Ctrl, *n* = 17; Cdc5L siRNA, *n* = 21) after release from IBMX, then stained for Bub3 (red) and DAPI (blue). Scale bars: 5 μm.

In mouse oocytes, APC/C mediated destruction of CCNB1 and securin to promote metaphasetoanaphase transition. We detected the endogenous CCNB1 expression at 9.5 h during meiotic maturation and found a higher level of CCNB1 expression in Cdc5L siRNA oocytes compared with control oocytes (Figure 4C). Furthermore, we monitored the activity of APC/C by the live-cell confocal system after low concentration of *Ccnb1* mRNA injection, and the result showed that the CCNB1 was rapidly degraded at the correct time (about from 10 h) in the control group, and stable expression of CCNB1 was observed in the Cdc5L siRNA group (Figure 5A and Supplementary Figure 2A). SAC is a well-known regulator of APC/C activity (Nilsson et al., 2008). To evaluate the SAC dynamic changes during meiosis I, we detected the SAC proteins, Bub3, in mouse oocytes by immunofluorescence staining. The GV stage oocytes were collected from female mice at 8 weeks of age, then the oocytes were cultured in an M2 medium containing 200-μM IBMX for 24 h and continuously cultured in IBMX-free M2 medium for 6 and 9.5 h. According to chromosome spreading, specific signals for Bub3 were detected on chromosome kinetochores in both Cdc5L knockdown oocytes and control oocytes at 6 h and disappeared in both groups at 9.5 h (Figure 5B and Supplementary Figure 2B). This result indicates that Cdc5L knockdown does not affect timely removal of SAC protein from kinetochores and that failure of APC/C activation in Cdc5L knockdown oocytes is independent of SAC activity.

Furthermore, we found that the PBE rate was significantly higher in Cdc5L overexpression oocytes at 9 and 10 h compared with control oocytes (Supplementary Figure 3A). We also found that the expression levels of SMC3 and securin were significantly decreased in the Cdc5L overexpression group compared with the control group at 10 h (Supplementary Figure 3B). These results indicated no effective APC/C activity for CCNB1 and securin degradation in Cdc5L siRNA oocytes, and Cdc5L is critical for the full activation of APC/C to induce the metaphase–anaphase I transition.

### Cell Division Cycle 5-Like Interacts With Securin Directly and Decreased Polar Body Extrusion Rate Can Be Rescued by Securin Knockdown

**FIGURE 6:**
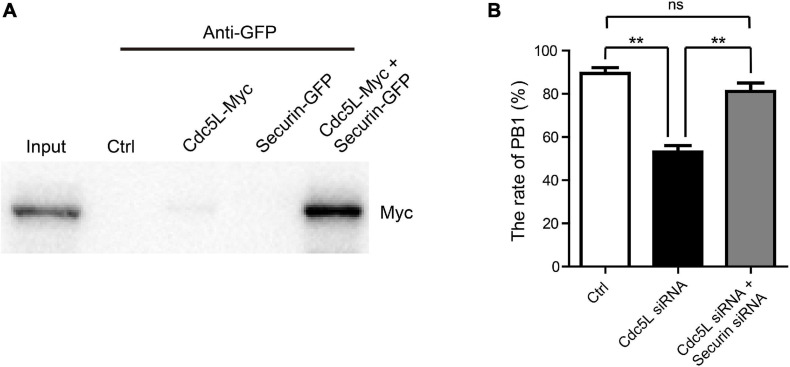
Cdc5L affects metaphase–anaphase I transition by acting through securin. **(A)** Representation of IP showing interaction between Cdc5L and securin. IP of EGFP-securin was performed with anti-GFP antibody, and precipitates were analyzed by Western blot using an anti-Myc antibody. Myc-Cdc5L and EGFP-securin were co-expressed in 293T cells (lane 5). Wild-type 293T cells (lane 2), 293T cells expressing Myc-Cdc5L alone (lane 3), and 293T cells expressing EGFP-securin alone (lane 4) were used as controls. Proteins of cells co-expressing Myc-Cdc5L and EGFP-securin were loaded as an input (lane 1). **(B)** Rate of PB1 extrusion was rescued by securin siRNA injection in Cdc5L knockdown oocytes. Ctrl, control siRNA injected (*n* = 86). Cdc5L siRNA, Cdc5L siRNA injected (*n* = 74). Cdc5L siRNA and securin siRNA, Cdc5L siRNA and securin siRNA co-injected (*n* = 77). Data are shown as means ± SEM. “*” represents *P* < 0.05; “**” represents *P* < 0.01. At least three replications were conducted.

Based on the results, securin is stably expressed in Cdc5L knockdown oocytes during meiosis I. To ask whether Cdc5L might directly interact with securin in oocytes, myc-labeled *Cdc5L* and EGFP-labeled *Securin* were co-transfected in 293T cells for IP. It was showed that Cdc5L clearly immunoprecipitated with securin (Figure 6A). To investigate whether knockdown of securin protein could recover the metaphase I arrest in Cdc5L knockdown oocytes, we injected both *securin* siRNA and *Cdc5L* siRNA or negative control siRNA into GV oocytes. The result showed that knockdown of securin in Cdc5L downregulated oocytes increased the rate of PB1 extrusion compared with the Cdc5L siRNA group, and there was no significant difference between the control group and both securin and Cdc5L siRNA injected group (Figure 6B). These data suggest that Cdc5L may directly interact with securin to control its degradation, which in turn plays an indispensable role in the metaphasetoanaphase transition during mouse oocyte meiotic maturation.

## Discussion

In mammalian species, the decreased oocyte quality and impaired oocyte maturation result in poor reproductive outcomes (Keefe et al., 2015; Gilchrist et al., 2016). Over the past few decades, although many studies have shed light on the understanding of oocyte meiotic maturation regulation, the overall network remains poorly understood. Here, we show that Cdc5L plays a critical role in regulating meiotic metaphase-to-anaphase transition and homologous chromosome separation in the first meiosis of oocyte by regulating APC/C–securin–separase–cohesin cascade, independent of SAC.

Several previous studies reported that Cdc5L has a critical role in regulating the mitotic cell cycle (Zhang et al., 2009; Mu et al., 2014; Qiu et al., 2016). Here, we provide evidence for a novel role of Cdc5L in regulating the mouse oocyte meiotic progression, which sharply differs from the role of Cdc5L in mitosis. In mitosis, the depletion of Cdc5L resulted in chromosome misalignment, kinetochore-microtubule attachment defects, and DNA damage, which finally led to mitotic catastrophe in HeLa cells (Mu et al., 2014). In our study, we proved that Cdc5L was a crucial regulator during the metaphase–anaphase I transition in mouse oocytes. The rate of PB1 extrusion was significantly reduced in Cdc5L knockdown oocytes. Knockdown of Cdc5L did not affect oocyte GVBD, spindle assembly, and chromosome alignment but arrested oocytes at the metaphase I after an extended period of culture. Chromosome spread showed that homologous chromosomes were not separated in Cdc5L knockdown oocytes failing to complete maturation even after extended culture. Both separase sensor mRNA injection and Smc3 staining showed that separase was not activated in Cdc5L knockdown oocytes.

It is well known that securin, whose degradation is controlled by APC/C, is the inhibitor of separase activity. The activity of APC/C is regulated by multiple upstream regulators, such as CDK1, CDC20, Cdh1, and cyclins (Yamamuro et al., 2008; Jin et al., 2010; Li et al., 2019). In our current study, we showed that securin or CCNB1 was timely degraded before metaphase-to-anaphase transition, accompanied by SAC inactivation. However, we found that although SAC was inactive, most oocytes were arrested at the metaphase I stage after Cdc5L knockdown, and both securin and cyclin B1 were not degraded, suggesting that Cdc5L is functional in regulating metaphase–anaphase I transition in the mouse oocyte and that Cdc5L is necessary for activation of APC/C independent of SAC. Strikingly, the knockdown of securin released the metaphase I arrest in Cdc5L knockdown oocytes. All these data suggest that Cdc5L probably acts as an activator of APC/C and is functional in the degradation of securin during oocyte meiotic maturation.

A previous study showed that Cdc5Lis the main component of putative E3 ubiquitin ligase complex (Zhang et al., 2009), and considering that, we speculated that Cdc5L might directly or indirectly regulate the activity of APC/C and combined with securin to prompt the degradation of securin. Interestingly, we found that Cdc5L can directly interact with securin, as revealed by IP assay. How these two proteins interact with each other and the relationship among Cdc5L, APC/C, and securin needs further experimental evidence to elucidate. How the Cdc5L regulates the process needs to be further investigated by the conditional knockout mouse model.

In summary, our work points to a novel function of Cdc5L in the metaphase–anaphase I transition of meiotic oocytes *via* regulating APC/C activity and degradation of securin. Our study has broad implications for understanding the oocyte meiotic signaling network in general.

## Data Availability Statement

The original contributions presented in the study are included in the article/Supplementary Material, further inquiries can be directed to the corresponding author/s.

## Ethics Statement

The animal study was reviewed and approved by the Institutional Animal Care Committee of Institute of Zoology, Chinese Academy of Sciences.

## Author Contributions

Q-YS, W-PQ, and H-YZ designed the experiments. JL constructed the plasmids of separase sensor and Securin-mcherry. Y-CO performed the GV oocyte injections. H-YZ performed the experiments and analyzed the data with the help of T-GM, C-HZ, and WY. Q-YS provided insightful suggestions for the manuscript and preparation. H-YZ wrote the manuscript with the help of other authors. All authors contributed to the article and approved the submitted version.

## Conflict of Interest

The authors declare that the research was conducted in the absence of any commercial or financial relationships that could be construed as a potential conflict of interest.

## Supplementary Material

Supplementary Figure 1 Immunofluorescence staining of spindle assembly and chromosome alignment after culture for 8 h following release from IBMX in both control and Cdc5L siRNA injected oocytes. The oocytes were analyzed for each group as follows: Ctrl, *n* = 16; Cdc5L siRNA, *n* = 17.

Supplementary Figure 2 Representative fluorescence intensity for CCNB1 and Bub3. **(A)** The fluorescence intensity of CCNB1 was analyzed in both control and Cdc5L siRNA oocytes. Data are shown as means ± SEM. “*” represents *P* < 0.05; “**” represents *P* < 0.01. At least three replications were conducted. **(B)** The fluorescence intensity of Bub3 was analyzed in both control and Cdc5L siRNA oocytes. Data are shown as means ± SEM. “*” represents *P* < 0.05; “**” represents *P* < 0.01. At least three replications were conducted.

Supplementary Figure 3 Overexpression of Cdc5L mRNA accelerated metaphase-to-anaphase during meiosis I. **(A)** Percentages of PBE were quantified in the control and Cdc5L mRNA oocytes. Data are shown as means ± SEM. “*” represents *P* < 0.05; “**” represents *P* < 0.01. At least three replications were conducted. The oocytes were analyzed for each group as follow: Ctrl, *n* = 128; Cdc5L mRNA, *n* = 117. **(B)** Expression of SMC3 and Securin protein as revealed by Western blotting analysis. The oocytes were collected 9.5 h after release from IBMX (150 oocytes per sample). At least three replications were conducted and 450 oocytes were used for each group. Data are shown as means ± SEM. “*” represents *P* < 0.05; “**” represents *P* < 0.01.

Supplementary Movie 1 Representative time-lapse fluorescence measurement of securin in control and Cdc5L siRNA group. **(A)** The securin-mCherry mRNA was expressed in control oocytes. Scale bar, 50 μm. **(B)** The securin-mCherry mRNA was expressed in Cdc5L siRNA oocytes. Scale bar, 50 μm.
