# Supplementary figures and images for: Cell Division Cycle 5-Like Regulates Metaphase-to-Anaphase Transition in Meiotic Oocyte
Source: Front Cell Dev Biol. 2021 Jul 1;9:671685. doi: 10.3389/fcell.2021.671685 (PMC8282184; doi:10.3389/fcell.2021.671685)

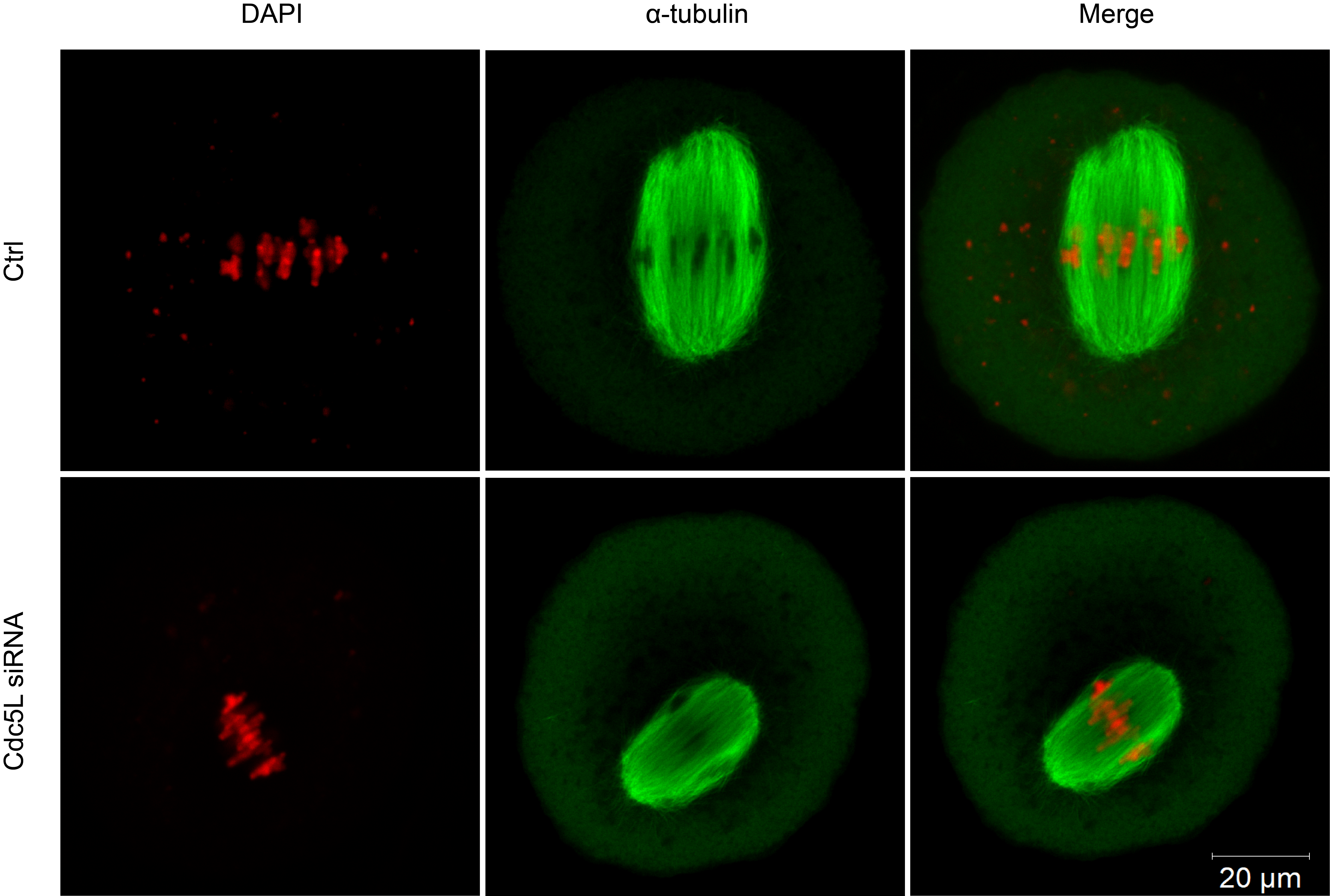

Supplement: Supplementary Figure 1 — Immunofluorescence staining of spindle assembly and chromosome alignment after culture for 8 h following release from IBMX in both control and Cdc5L siRNA injected oocytes. The oocytes were analyzed for each group as follows: Ctrl, n = 16; Cdc5L siRNA, n = 17. [file Image_1.tif]

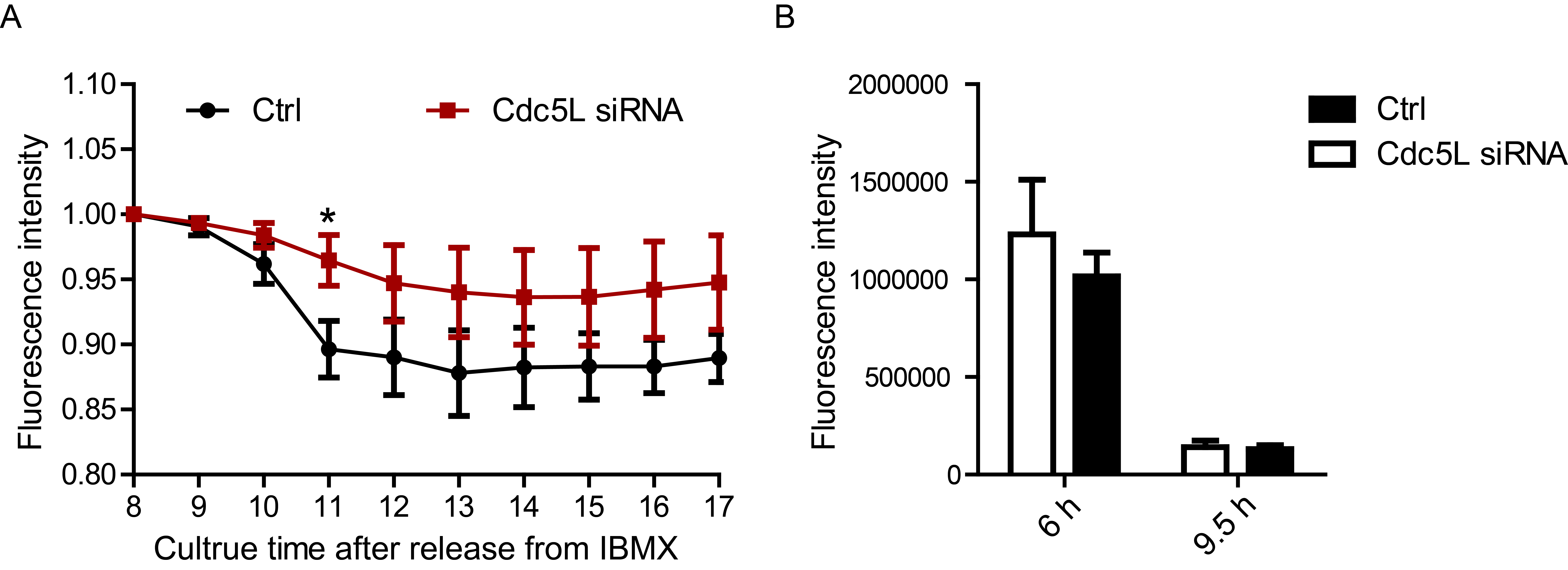

Supplement: Supplementary Figure 2 — Representative fluorescence intensity for CCNB1 and Bub3. (A) The fluorescence intensity of CCNB1 was analyzed in both control and Cdc5L siRNA oocytes. Data are shown as means ± SEM. “*” represents P < 0.05; “**” represents P < 0.01. At least three replications were conducted. (B) The fluorescence intensity of Bub3 was analyzed in both control and Cdc5L siRNA oocytes. Data are shown as means ± SEM. “*” represents P < 0.05; “**” represents P < 0.01. At least three replications were conducted. [file Image_2.tif]

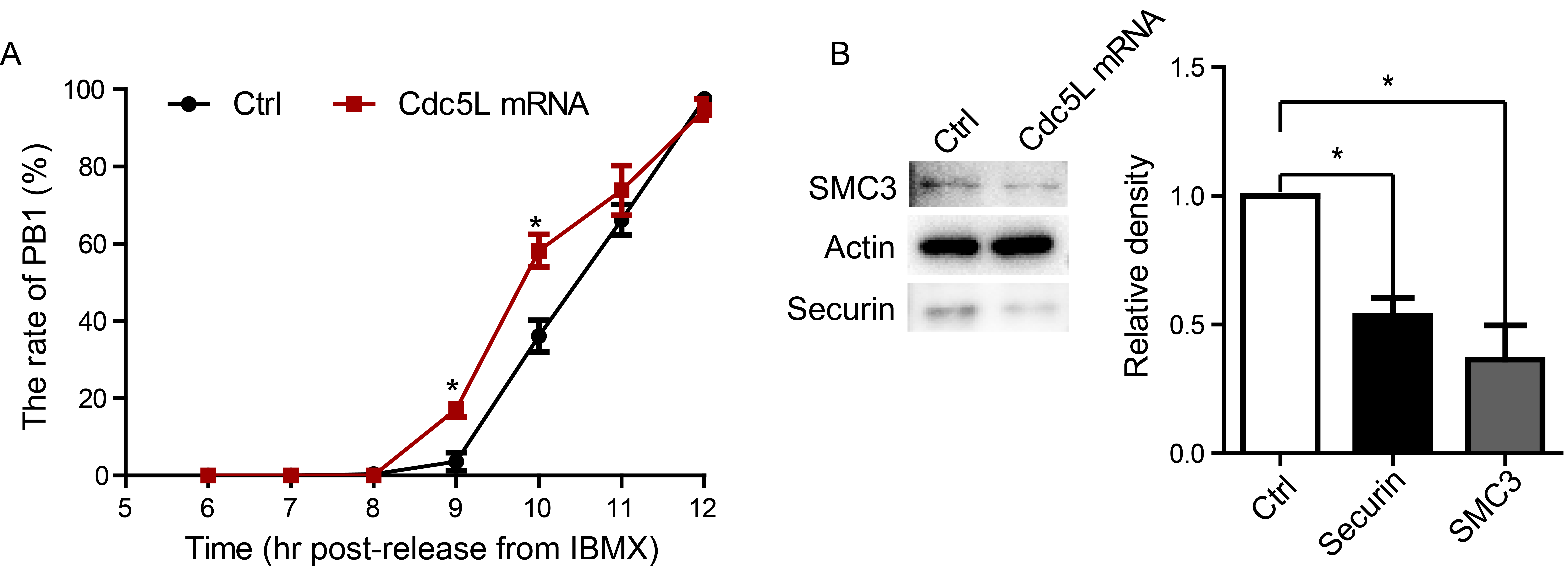

Supplement: Supplementary Figure 3 — Overexpression of Cdc5L mRNA accelerated metaphase-to-anaphase during meiosis I. (A) Percentages of PBE were quantified in the control and Cdc5L mRNA oocytes. Data are shown as means ± SEM. “*” represents P < 0.05; “**” represents P < 0.01. At least three replications were conducted. The oocytes were analyzed for each group as follow: Ctrl, n = 128; Cdc5L mRNA, n = 117. (B) Expression of SMC3 and Securin protein as revealed by Western blotting analysis. The oocytes were collected 9.5 h after release from IBMX (150 oocytes per sample). At least three replications were conducted and 450 oocytes were used for each group. Data are shown as means ± SEM. “*” represents P < 0.05; “**” represents P < 0.01. [file Image_3.tif]
